# Rhadinovirus Host Entry by Co-operative Infection

**DOI:** 10.1371/journal.ppat.1004761

**Published:** 2015-03-19

**Authors:** Clara Lawler, Ricardo Milho, Janet S. May, Philip G. Stevenson

**Affiliations:** 1 Sir Albert Sakzewski Virus Research Centre, School of Chemistry and Molecular Biosciences, Royal Children’s Hospital and University of Queensland, Brisbane, Australia; 2 Division of Virology, Department of Pathology, University of Cambridge, Cambridge, United Kingdom; Louisiana State University Health Sciences Center, UNITED STATES

## Abstract

Rhadinoviruses establish chronic infections of clinical and economic importance. Several show respiratory transmission and cause lung pathologies. We used Murid Herpesvirus-4 (MuHV-4) to understand how rhadinovirus lung infection might work. A primary epithelial or B cell infection often is assumed. MuHV-4 targeted instead alveolar macrophages, and their depletion reduced markedly host entry. While host entry was efficient, alveolar macrophages lacked heparan - an important rhadinovirus binding target - and were infected poorly *ex vivo*. *In situ* analysis revealed that virions bound initially not to macrophages but to heparan^+^ type 1 alveolar epithelial cells (AECs). Although epithelial cell lines endocytose MuHV-4 readily *in vitro*, AECs did not. Rather bound virions were acquired by macrophages; epithelial infection occurred only later. Thus, host entry was co-operative - virion binding to epithelial cells licensed macrophage infection, and this in turn licensed AEC infection. An antibody block of epithelial cell binding failed to block host entry: opsonization provided merely another route to macrophages. By contrast an antibody block of membrane fusion was effective. Therefore co-operative infection extended viral tropism beyond the normal paradigm of a target cell infected readily *in vitro*; and macrophage involvement in host entry required neutralization to act down-stream of cell binding.

## Author Summary

All viral infections start with host entry. Entry into cells is studied widely in isolated cultures; entry into live hosts is more complicated and less well understood: our tissues have specific anatomical structures and our cells differ markedly from most cultured cells in size, shape and behaviour. The respiratory tract is a common site of virus infection. Size dictates where inhaled particles come to rest, and virus-sized particles can reach the lungs. Rhadinoviruses chronically infect both humans and economically important animals, and cause lung disease. We used a well-characterized murine example to determine how a rhadinovirus enters the lungs. At its peak, infection was prominent in epithelial cells lining the lung air spaces. However it started in macrophages, which normally clear the lungs of inhaled debris. Only epithelial cells expressed the molecules required for virus binding, but only macrophages internalized virus particles after binding; infection involved interaction between these different cell types. Blocking epithelial infection with an antibody did not stop host entry because attached antibodies increase virus uptake by lung macrophages; but an antibody that blocks macrophage infection was effective. Thus, understanding how rhadinovirus infections work in normal tissues provided important information for their control.

## Introduction

The γ-herpesviruses chronically infect most mammals and cause disease in humans [1] and economically important ungulates [2]. Vaccines have failed to prevent infection. In part this reflects that host entry is ill-understood. Oral entry is often assumed, because the archetypal Epstein-Barr virus (EBV) causes acute tonsillitis. However clinical presentation occurs at least 1 month after EBV acquisition [3] and correlates better with peak salivary shedding. Thus, it may reflect host exit rather than entry, and because virus shedding involves reactivation from circulating B cells [4] entry and exit routes need not coincide.

Lymphocryptoviruses such as EBV are known only in primates; Rhadinoviruses such as the Kaposi’s Sarcoma-associated Herpesvirus (KSHV) are more widespread [5]. KSHV in nasal secretions [6], and pulmonary Kaposi’s Sarcoma complicating AIDS [7], suggest respiratory infection. Consistent with this idea, Ovine herpesvirus-2 and Alcelaphine herpesvirus-1 show salivary and nasal shedding, and infect in nebulized form [8]; Equid γHV-5 causes respiratory disease [9] and can be isolated from the respiratory tract [10]; and Bovine herpesvirus-4 colonizes the respiratory tract [11], and persists in calves after intranasal (i.n.) inoculation [12].

MuHV-4 (strain MHV-68) [13, 14] allows rhadinovirus infection to be analysed in mice. Like EBV and KSHV, it is B cell tropic. MuHV-4 infects readily mice i.n., and infects orally only if it spills into the respiratory tract [15]. When given i.n. under anesthesia it infects the lungs [16]; without anesthesia it infects the upper respiratory tract [17]; and it transmits sexually from female mice to co-caged males [18]. How MuHV-4 transmits in its natural host of yellow-necked mice [19] is unknown. Analysis of wood mice identified DNA from MuHV-4 or a related virus in lungs [20], and lung inoculation is used widely for experimental infections. Therefore MuHV-4 is suited to analyzing this mode of host entry.

A key unknown is the first infected cell type. Lungs contain type 1 AECs, which comprise 95% of the alveolar surface area; type 2 AECs, which secrete surfactant; macrophages, which phagocytose inhaled debris [21]; and some lymphocytes. By 5 days after i.n. inoculation, MuHV-4 antigens are detectable in type 1 AECs and large mononuclear cells [16]. DNA of i.n.-inoculated, replication-deficient MuHV-4 has been detected by PCR of B cells from lung homogenates [22, 23], suggesting that they are a primary infection target. However MuHV-4 normally binds to heparan, which B cells express poorly [24, 25], and shows a post-binding block to B cell infection that is not overcome until virions pass through myeloid cells [26]. PCR detects replication-deficient MuHV-4 DNA also in association with B cells recovered from splenic homogenates after intraperitoneal (i.p.) inoculation [22, 23], when *in situ* analysis shows no evidence of B cell infection [27]. Therefore the PCR signals associated with lung B cells might reflect adsorbed inoculum debris rather than infection.

Any understanding of MuHV-4 infection must encompass its dependence on heparan for cell binding [24], a characteristic common to many rhadinoviruses [28–30]. The MuHV-4 gp70 and gH/gL bind to heparan [31, 32], and gp150 further inhibits heparan-independent binding until heparan is engaged [24]. Thus, gL^-^gp70^-^ MuHV-4 infects poorly both *in vitro* and *in vivo*, unless gp150 is disrupted also [33]. The Bovine Herpesvirus-4 gp150 homolog, gp180, is functionally similar [34]. While most transformed epithelial cells express abundant heparan, most *in vivo* epithelia do so only basolaterally [35]. Thus, heparan-dependent host entry by incoming, apical virions is not necessarily straightforward. The olfactory neuroepithelium, which we have analysed previously [17], unusually expresses both basolateral and apical heparan. The genital epithelium lacks apical heparan [36]; entry here may depend on epithelial trauma, as MuHV-4-infected cells are seen underneath rather than in the stratified squamous epithelium [18]. We asked which cell type MuHV-4 targets in the lungs and how this relates to heparan binding. Our aim was to understand better rhadinovirus entry into new hosts and so establish a rational basis for infection control.

## Results

### Viral lytic antigens appear in lung myeloid cells before epithelial cells

**Fig 1:**
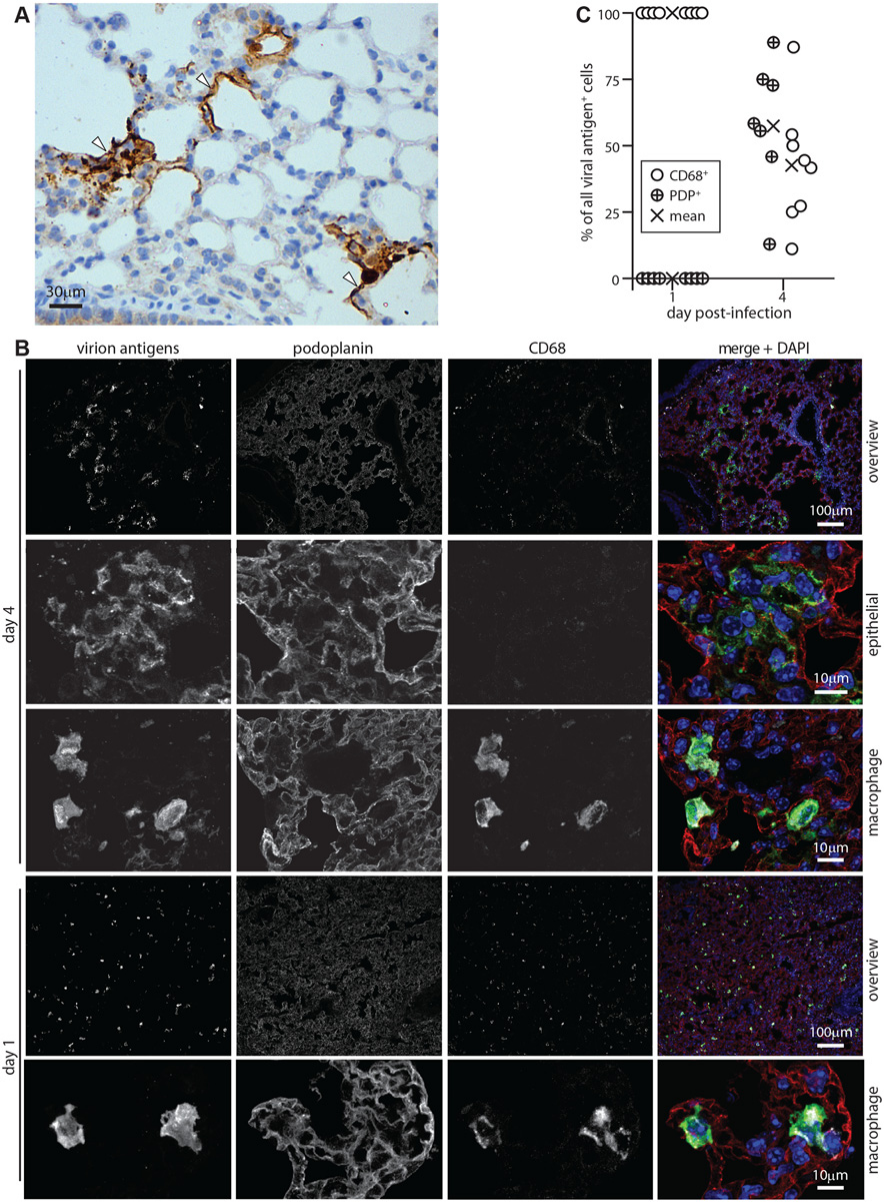
Lytic antigen expression in peak and early MuHV-4 lung infections. **A** C57BL/6 mice were given i.n. MuHV-4 (10^4^ p.f.u.). After 4 days lung sections were stained for viral antigens with a polyclonal rabbit serum (brown) and co-stained with hematoxylin (blue). Arrows show infected cells with characteristic type 1 AEC morphology. **B** C57BL/6 mice were given i.n. MuHV-4 (10^5^ p.f.u.). At early (day 1) or peak (day 4 for this infection dose) infection, lung sections were stained for viral lytic antigens (green in merge), PDP (type 1 AECs, red in merge) and CD68 (macrophages, white in merge). Nuclei were stained with DAPI (blue in merge). Higher magnification day 4 images show example viral antigen^+^ PDP^+^ and CD68^+^ cells. At day 1 only CD68^+^ cells were viral antigen^+^. These are smaller than AECs, so the overview has more punctate staining. **C** Counts of CD68^+^ and PDP^+^ viral antigen^+^ cells on lung sections. Circles show individual mice, crosses show means. Each point corresponds to >50 cells. At day 4 significantly more viral antigen^+^ cells were PDP^+^ and significantly fewer CD68^+^ than at day 1 (p<0.001 by Student’s unpaired 2 tailed t test).

Four days after i.n. MuHV-4 inoculation, viral antigens were expressed in morphologically typical type 1 AECs, whose extensive cytoplasmic projections line the lung air spaces (Fig. 1A). This tropism was confirmed by co-staining lungs for viral antigens and the type 1 AEC marker podoplanin (PDP) [37] (Fig. 1B)—at 4 days post-inoculation >50% of viral antigen^+^ cells were PDP^+^ (Fig. 1C). The morphologically distinct remainder expressed the macrophage / granulocyte marker CD68 [38–40]. However at 1 day post-inoculation viral antigens were confined to CD68^+^ cells. Therefore while infection spread to type 1 AECs, it appeared to start in macrophages or granulocytes.

### Incoming virions infect alveolar macrophages

**Fig 2:**
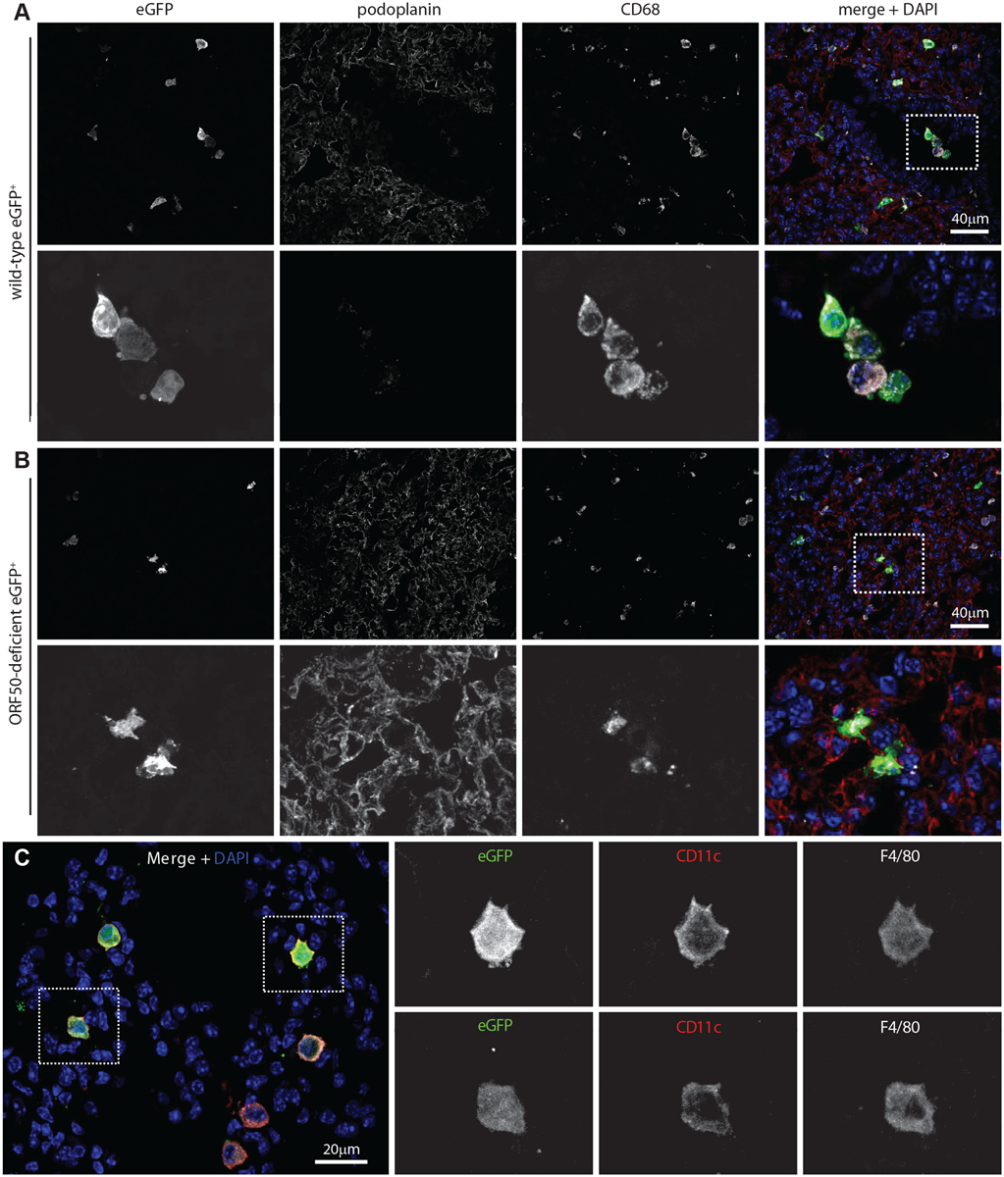
Viral eGFP expression in early lung infection. **A** Lungs of C57BL/6 mice 1 day after i.n. HCMV IE1-eGFP^+^ MuHV-4 (10^5^ p.f.u.) were stained for eGFP (green in merge), PDP (type 1 AECs, red in merge), and CD68 (alveolar macrophages, white in merge). Nuclei were stained with DAPI (blue in merge). All eGFP^+^ cells were CD68^+^. The images are representative of sections from 3 mice. The upper panels show overviews. The lower panels show the boxed region at higher magnification. **B** Lungs of mice given i.n. ORF50^-^ eGFP^+^ MuHV-4 (10^5^ p.f.u.), which expresses eGFP but no new lytic antigens, were stained as in A. Again no eGFP^+^ cells were PDP^+^. The upper panels show overviews. The lower panels show the boxed region at higher magnification. **C** Lung sections of mice infected as in **B** were stained for viral eGFP (green), CD11c (red) and F4/80 (white). Images are representative of sections from 3 mice. The right-hand panels show individual channels of the boxed regions at higher magnification.

MuHV-4-immune sera recognize mainly lytic antigens. As much early lung infection is latent [41], we tracked host entry more comprehensively via lytic cycle-independent [42] eGFP expression from a viral HCMV IE1 promoter (Fig. 2A). At 1 day post-inoculation, 238/238 eGFP^+^ cells on lung sections of 3 mice were CD68^+^. None was PDP^+^. MuHV-4 rendered incapable of uncomplemented lytic spread by disruption of its ORF50 transactivator (ORF50^-^) showed a similar eGFP distribution: 78/78 eGFP^+^ cells on lung sections from 5 mice expressed CD68, and none expressed PDP (Fig. 2B). Therefore CD68^+^ cells were direct infection targets. More than 80% of eGFP^+^ cells expressed also the tissue macrophage marker F4/80 [39] and the alveolar macrophage / dendritic cell marker CD11c [38, 40] (Fig. 2C). Macrophage populations are heterogeneous and often not clearly demarcated [38], but combined CD11c, CD68 and F4/80 expression argued that most infected cells were alveolar macrophages.

The lack of HCMV IE1-driven eGFP expression in type 1 AECs at day 1 post-inoculation established that they were uninfected rather than just slow to initiate lytic infection. We saw also no eGFP expression in B cells at day 1 by either eGFP^+^ wild-type (WT, >100 eGFP^+^ cells counted from 3 mice) or eGFP^+^ORF50^-^ MuHV-4 (>50 eGFP^+^ cells counted from 3 mice) (S1 Fig). Nor did MuHV-4 with EF1α promoter-driven eGFP expression show a day 1 infection of B220^+^ lung B cells or type 1 AECs (S1 Fig, >100 eGFP^+^ cells counted from 3 mice). Therefore incoming virions targeted selectively alveolar macrophages.

### Genetic tagging shows early virus replication in myeloid cells

Viral lytic gene expression suggests but does not prove lytic replication. To determine how alveolar macrophage infection contributed to virus production we infected lysM-cre mice with MuHV-4 in which cre-mediated recombination switches fluorochrome expression from mCherry^+^ (red) to eGFP^+^ (green) (MHV-RG) [26]. LysM-cre mice express cre from the myeloid lysozyme locus [43]. To identify cre^+^ lung cells we crossed them with a reporter strain in which cre activates Zsgreen expression from a widely expressed promoter [44]. PDP^+^ cells lacked Zsgreen (S2 Fig). CD68^+^ cells expressed Zsgreen in an endosomal distribution. Type 2 AECs (surfactant protein C precursor^+^, CD68^-^, MAC-2^-^) also expressed Zsgreen—in a uniform rather than endosomal distribution. However in C57BL/6 mice given HCMV-IE1-eGFP^+^ MuHV-4 they remained eGFP^-^ (S3 Fig) and so were not infected. Thioglycollate-induced Gr-1^+^ peritoneal exudate neutrophils of lysM-cre x Zsgreen mice were Zsgreen^+^, consistent with published data [43], but lung cells expressing neutrophil markers (Gr-1 or myeloperoxidase) were not (S2 Fig). They also lacked eGFP expression by HCMV-IE1-eGFP^+^ MuHV-4. Therefore we interpreted MHV-RG switching in lysM-cre mice as an infection of alveolar macrophages. Some switching in lung dendritic cells was possible, but they express little lysM [43] or CD68 [39], so there was no other indication that dendritic cells were a significant target for incoming virions.

**Fig 3:**
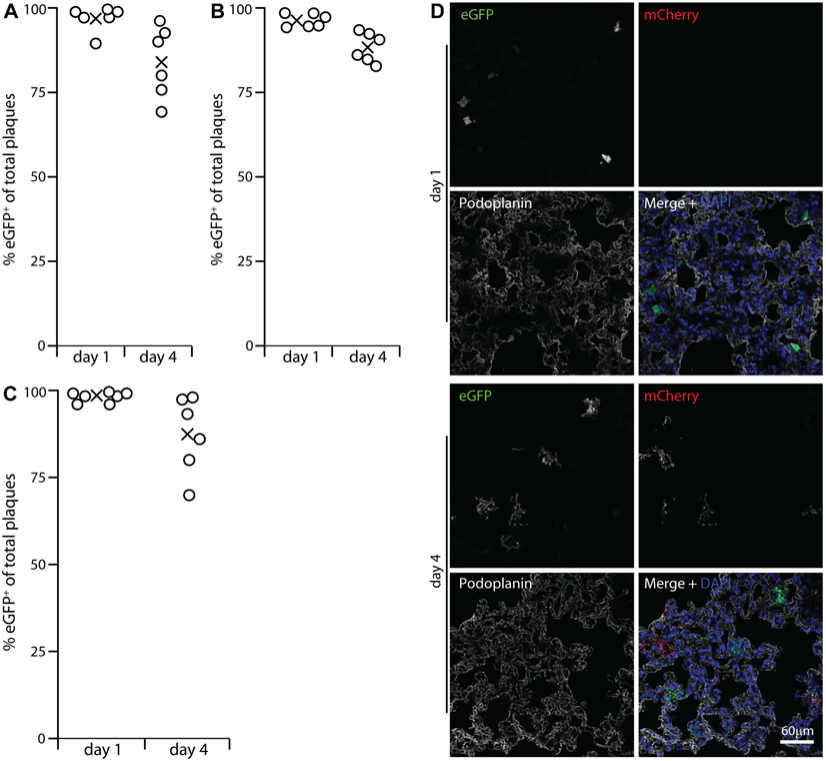
Functional evidence for virus replication in lysM^+^ and CD11c^+^ cells. **A** LysM-cre mice were given i.n. MuHV-4 in which cre switches fluorochrome expression from mCherry to eGFP (MHV-RG). 1 and 4 days later, viruses recovered from lungs were typed as mCherry^+^ or eGFP^+^. None was eGFP^+^mCherry^+^or eGFP^-^mCherry^-^. %eGFP^+^ gives the proportion of viruses exposed to cre. The input virus was 0% eGFP^+^. Circles show individual mice, crosses show means. **B** A repeat experiment showed the same high recombination at both time points, but higher at day 1 than day 4. Across both trials the % eGFP^+^ was significantly higher at day 1 than at day 4 (p<0.002 by Student's 2-tailed unpaired t test). **C** Lung sections of mice infected as in A were stained for eGFP (green in merge), mCherry (red in merge) and PDP (white in merge). Nuclei were stained with DAPI (blue). At day 1 all fluorescent cells were PDP^-^ and morphologically lung macrophages. Of these >90% were eGFP^+^. At day 4 there were both eGFP^+^ and mCherry^+^ PDP^+^ cells. Images are presentative of 12 sections from 3 mice. **D** CD11c-cre mice, which express cre in alveolar macrophages, were infected with MHV-RG as in A. Most recovered virus showed evidence of exposure to cre, again with more switching at day 1 than at day 4 (p<0.03 by Student's 2-tailed unpaired t test).

The MHV-RG recovered from lysM-cre lungs at 1 day post-inoculation was >96% eGFP^+^, indicating that essentially all of it had passed through an alveolar macrophage (Fig. 3A). Surprisingly the MHV-RG recovered after 4 days showed only 85% switching. This reduction in % switched virus with time was consistent and statistically significant (Fig. 3B), suggesting that virions could also enter the lungs without infecting a macrophage, but that virus production was then delayed. We confirmed myeloid infection as the predominant route by infecting CD11c-cre mice (Fig. 3C), as alveolar macrophages (and dendritic cells) are CD11c^+^ [38]. Again the virus recovered from lungs after 1 day was >96% eGFP^+^, and that recovered after 4 days was >85% eGFP^+^.

The prominent viral lytic antigen expression in type 1 AECs at day 4 post-inoculation (Fig. 1) but not day 1 implied that they receive and amplify virus from alveolar macrophages. Their lack of HCMV IE1-driven or EF1α-driven eGFP expression at day 1 (Figs. 2, S1) excluded acute infection. Day 1 MHV-RG-infected lung sections (Fig. 3D) confirmed this: >95% of fluorescent cells were morphologically typical alveolar macrophages and PDP^-^. Of these 92.6 ± 4.3% were eGFP^+^ (mean ± SD of counts from 8 mice), confirming efficient viral fluorochrome switching in infected myeloid cells. However at day 4, when 63.7% of fluorescent cells were PDP^+^, 48.5 ± 10.6% of these were eGFP^-^mCherry^+^ (mean ± SD of counts from 8 mice). Thus, not all type 1 AEC infection was down-stream of myeloid cell infection. And while no eGFP^+^ cells were PDP^+^ 1 day after i.n. eGFP^+^ORF50^-^ MuHV-4 inoculation of C57BL/6 mice (Fig. 2), 35.5 ± 29.7% of eGFP^+^ cells were PDP^+^ after 5 days (mean ± SD, 6 sections from 3 mice) (S4 Fig). Therefore a delayed type 1 AEC infection without prior macrophage infection seemed to account for the reduction in % virus switching between days 1 and 4 in Fig. 3A-3C. Either virions eventually penetrated AECs, or macrophages licenced AEC infection without becoming infected themselves, for example by recycling virions from early endosomes to exosomes and releasing them in a licensed form [45].

### Alveolar macrophage depletion compromises host entry

**Fig 4:**
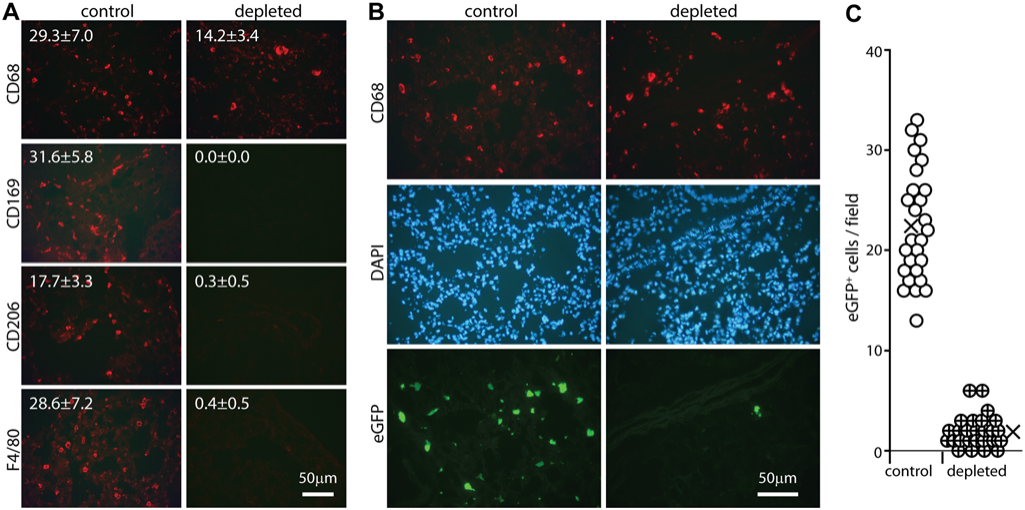
Effect of macrophage depletion on host entry. **A** CD169^+/DTR^ mice were given i.p. diphtheria toxin (500ng) or not daily for 2 days, and lung sections stained for macrophage markers. Representative images are shown. Numbers show mean ± SD of cell counts per field of view on 9 sections from 3 mice. The reductions with depletion were all highly significant (p<0.001 by Student’s unpaired 2 tailed t test). **B** Mice depleted of CD169^+^ cells as in **A** were infected i.n. with ORF50^-^eGFP^+^ MuHV-4 (10^5^ p.f.u.). After 1 day lung sections were stained for CD68 and analysed for infection by staining for eGFP. Nuclei were stained with DAPI. Representative images are shown. **C** Quantitation of eGFP numbers from mice treated as in **B** (3 fields of view per section on 9 sections from 3 mice) showed that toxin treatment reduced significantly lung infection (p<10^-5^ by Student's 2-tailed unpaired t test). Circles show individual counts, crosses show means.

Most lung macrophages express CD169 [46], so we used CD169-DTR mice [47] to test their importance for host entry (Fig. 4). Mice were given diphtheria toxin i.p. for 2 days, then given eGFP^+^ ORF50^-^ MuHV-4 i.n. for 1 day. Staining lungs for CD169, CD68, F4/80 and CD206 (Fig. 4A) showed that CD169 expression was ablated completely. CD206 and F4/80 expression were ablated >90%, consistent with most lung macrophages expressing CD169. EGFP^+^ cell numbers in toxin-treated mice were <10% those of untreated controls (Fig. 4B). CD68^+^ cells were still seen, but they were larger than in undepleted mice and lacked other alveolar macrophage markers. Such cells were not evident in undepleted infected mice, and so seemed likely to be bone marrow-derived monocytes or granulocytes, recruited in response to alveolar macrophage ablation. They were clearly poor infection targets. Therefore alveolar macrophages played a crucial role in virus acquisition, for which lineage-distinct bone marrow-derived myeloid cells [48] did not substitute.

### Alveolar and other macrophages are infected poorly *ex vivo*

**Fig 5:**
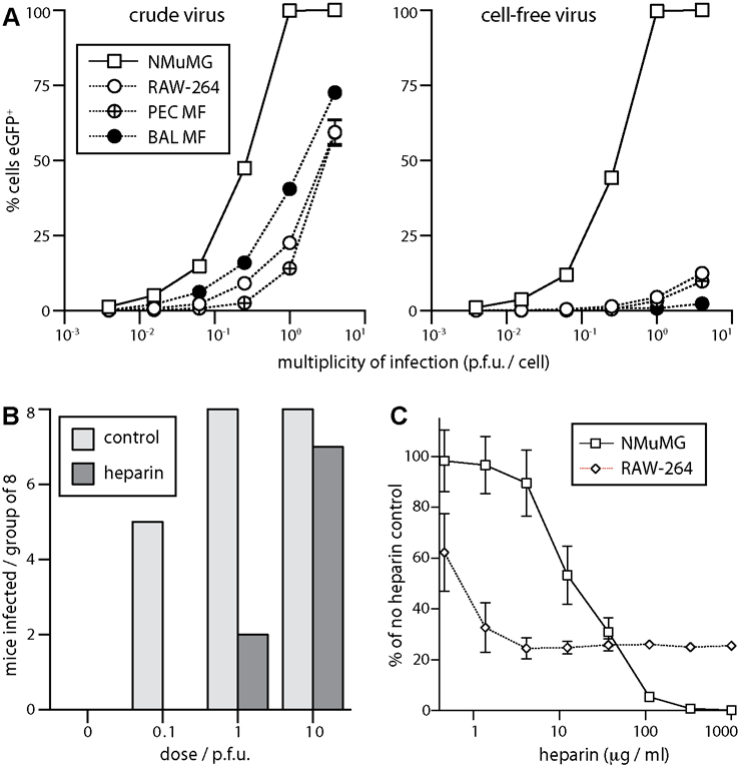
Sensitivity of host entry to inhibition by heparin. **A** EGFP^+^ MuHV-4 stocks harvested from infected cells + supernatants (crude virus), or from supernatants alone followed by 0.45μm filtration (cell-free virus), were used to infect NMuMG epithelial cells, RAW-264 monocytes, macrophages from peritoneal washes (PEC Mϕ), and alveolar macrophages from bronchial washes (BAL Mϕ). The cells were cultured overnight with phosphonoacetic acid (100μg/ml) to allow eGFP expression without further infection spread. EGFP^+^ cells were then identified by flow cytometry. Each point shows mean ± SD of triplicate cultures—most SDs are smaller than the symbols and so not visible. Crude virus infected significantly more macrophages (p<10^-4^ by Student’s unpaired 2-tailed t test) and an equivalent number of epithelial cells (p>0.2), indicating a specific barrier to macrophage infection by cell-free virions. **B** Cell-free eGFP^+^ MuHV-4 (10^4^ p.f.u./ml) was incubated with heparin (1mg/ml in DMEM, 2h, 23°C) or in DMEM alone (control), then given i.n. to C57BL/6 mice in 30μl at the doses shown (8 mice per group). After 14 days the mice were tested for infection by ELISA of MuHV-4-specific serum IgG. Incubation with heparin reduced significantly infection rates at 0.1 p.f.u. (p<0.03 by Fisher’s exact test) and 1 p.f.u. (p<0.01). **C** Cell-free eGFP^+^ MuHV-4 was incubated with different heparin concentrations (2h, 23°C) then added to NMuMG epithelial cells (0.5 p.f.u./cell) or RAW-264 monocytes (5 p.f.u./cell). EGFP expression was determined after overnight culture as in **A**. Each point shows mean ± SD of triplicate cultures. Virus without heparin gave 65.3% eGFP^+^ NMuMG cells and 13.1% eGFP^+^ RAW-264 cells. Heparin inhibited RAW-264 infection significantly better than NMuMG cell infection at <30μg/ml, but significantly worse at >100μg/ml (p<0.001 by Student’s 2-tailed unpaired t test, comparing % inhibition for each cell type).

Host entry via lung macrophages was unexpected, as MuHV-4 infects the lungs efficiently [49] and myeloid cells poorly [26]. The cell-free virions used for *in vivo* infection poorly infected *ex vivo* lung macrophages, peritoneal macrophages or the monocyte line RAW-264 (Fig. 5A). Virus stocks containing cell debris infected macrophages with reasonable efficiency. However cell-associated virus infections would occur only after host entry. Efficient alveolar macrophage infection by cell-free virions evidently depended on their normal tissue context.

MuHV-4 that lacks heparan binding due to gp70 and gL disruption infects mice i.n. 100-fold less well than the WT, unless its gp150, which inhibits heparan-independent binding, is disrupted too [33]. This result implies that host entry requires heparan engagement. We confirmed it by pre-incubating WT virions with soluble heparin (Fig. 5B). Seroconversion by virus-specific serum IgG ELISA—the the most sensitive measure of low dose infection in immunocompetent mice [33]—was reduced 10–100-fold by the heparin treatment. Pre-incubating virions with heparin also reduced NMuMG epithelial cell infection 100-fold (Fig. 5C). However, while low dose heparin inhibited RAW-264 monocyte infection better than NMuMG cell infection—presumably because RAW-264 cells have little heparan for virion binding to start with [26]—there was also a heparin-resistant component of infection, such that the maximal inhibition was only 4-fold. Therefore the efficiency of lung infection and its susceptibility to inhibition by heparin both suggested an initial interaction with epithelial cells rather than macrophages.

### Alveolar epithelial cells express heparan; alveolar macrophages do not

**Fig 6:**
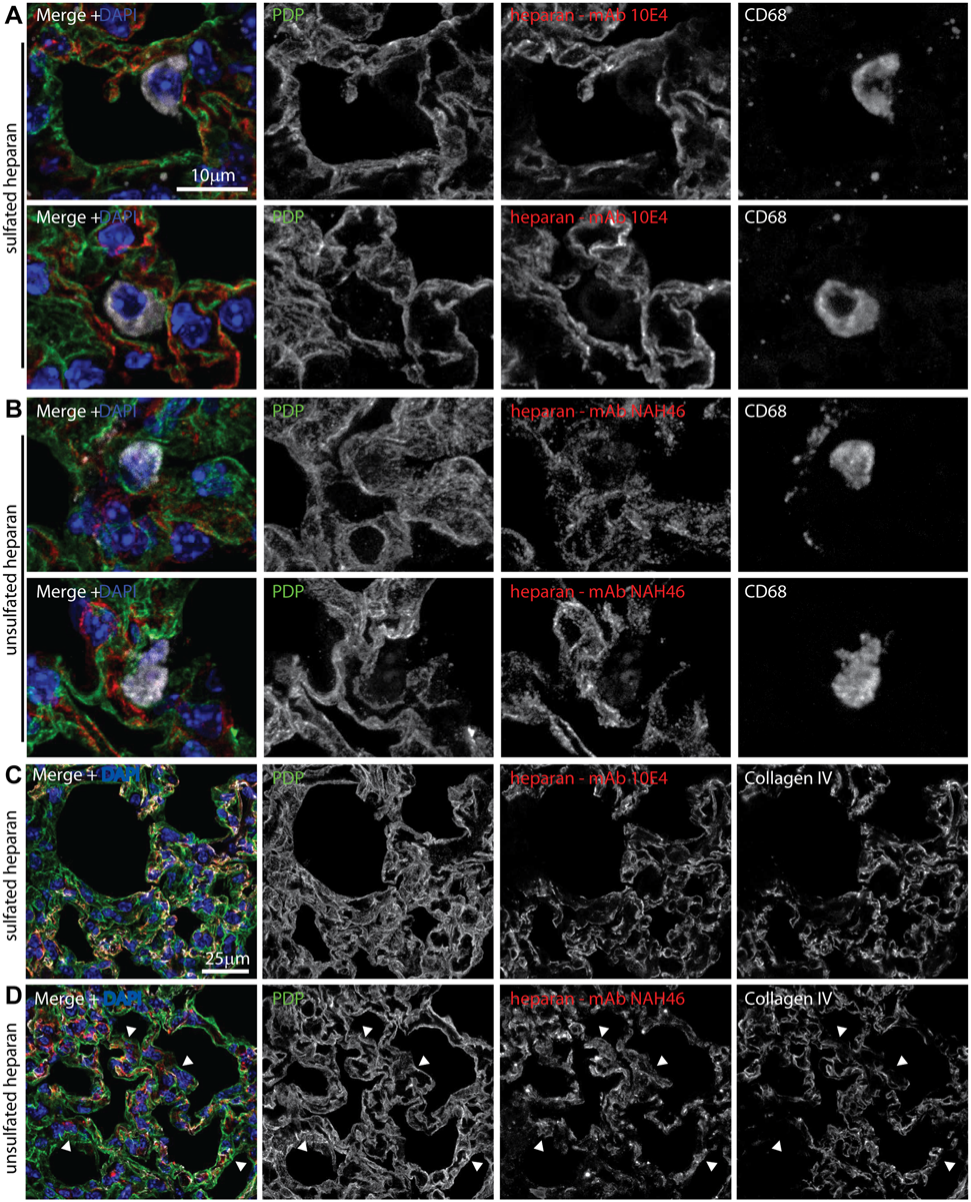
Heparan expression in lungs. **A** Lungs of naive mice were stained for PDP (green in merge), CD68 (white in merge), and sulfated heparan with mAb 10E4 (red in merge). Nuclei were stained with DAPI (blue). Two example images are shown, representative of 10 sections from 2 mice. Heparan co-localized with PDP (type 1 AECs) and not with CD68 (alveolar macrophages). **B** Lung sections as in a were stained for PDP, CD68, and unsulfated heparan with mAb NAH46 (red in merge). Two example images are shown, representative of 10 sections from 2 mice. Heparan co-localized with PDP and not with CD68. **C** Lung sections as in a were stained for PDP, collagen IV (white in merge), and sulfated heparan with mAb 10E4. Heparan and collagen IV showed equivalent distributions. Images are representative of 8 sections from 2 mice. **D** Lung sections as in a were stained for PDP, collagen IV, and unsulfated heparan with mAb NAH46. Again heparan and collagen IV showed extensive co-localization, but some NAH46^+^PDP^+^ regions were collagen IV^-^ (arrows). These abutted alveolar air spaces, and so correspond to the apical surface of type 1 AECs.

To identify heparan^+^ cells in the lungs we stained sections with mAb 10E4, which recognizes sulfated heparan [50] (Fig. 6A), and with mAb NAH46, which recognizes non-sulfated heparan [51] (Fig. 6B). Staining by both mAbs co-localized with PDP (type 1 AECs) and not with CD68 (alveolar macrophages). To distinguish apical from basolateral heparan, we co-stained sections for the alveolar basement membrane component collagen IV (Fig. 6C). Sulfated heparan (mAb 10E4) co-localized completely with collagen IV and so was just basolateral. Unsulfated heparan (mAb NAH46) also co-localized largely with collagen IV. However there were also NAH46^+^ surfaces abutting the air spaces that lacked collagen IV. Therefore specifically unsulfated heparan was accessible to incoming virions on the apical AEC surface.

### Incoming virions bind to type 1 AECs but accumulate in macrophages

**Fig 7:**
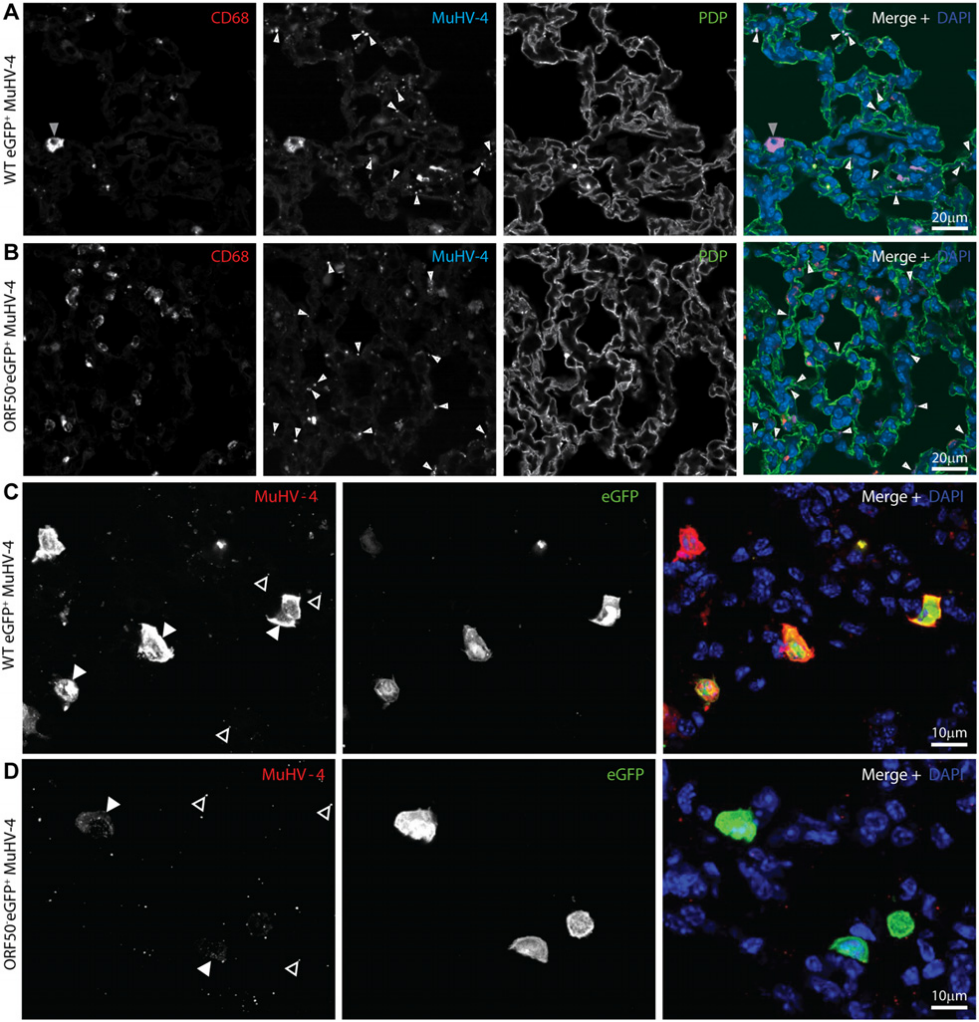
*In vivo* MuHV-4 binding and uptake. **A.** C57BL/6 mice were infected i.n. (10^5^ p.f.u.) with eGFP^+^ MuHV-4. 1 day later lung sections were immunostained for CD68 (red in merge), virion antigens (cyan in merge) and PDP (green in merge). Nuclei were stained with DAPI (blue in merge). Significantly more isolated antigen^+^ dots (virions, white arrows) associated with PDP^+^ AECs than with CD68^+^ macrophages (p<10^-6^ by Student’s 2 tailed unpaired t test); but more antigen^+^ cells (grey arrow) were macrophages (p<10^-6^). The images are representative of sections from 3 mice. **B.** C57BL/6 mice were infected i.n. (10^5^ p.f.u.) with ORF50^-^ eGFP^+^ MuHV-4. 1 day later lung sections were stained as in a. Again, significantly more isolated antigen^+^ dots (white arrows) associated with PDP^+^ AECs than with CD68^+^ macrophages (p<10^-6^ by Student’s 2 tailed unpaired t test). **C.** Lung sections of mice infected as in a were stained 1 day later for virion antigens (red in merge) and eGFP (green in merge). Nuclei were stained with DAPI (blue in merge). At this time only macrophages express new lytic antigens or viral eGFP (Figs. 1–2). Significantly more strong, diffuse lytic antigen staining (lytic infection, filled arrow) was in eGFP^+^ than in eGFP^-^ cells (p<10^-6^ by Student’s 2-tailed unpaired t test); but significantly more isolated antigen^+^ dots (open arrows) associated with eGFP^-^ cells (p<10^-6^). The images are representative of 9 sections from 3 mice. **d.** Mice were infected as in b. 1 day later lung sections were stained as in c. Input virion antigens (from input virions as this virus is replication-deficient, filled arrow) accumulated mainly in eGFP^+^ cells, whereas isolated antigen^+^ dots (open arrows) associated mainly with eGFP^-^ cells (p<10^-6^).

One day after WT MuHV-4 inoculation, viral antigens were evident as isolated dots—presumably virions. Of these 94.8 ± 3.9% associated with PDP^+^ type 1 AECs (>300 dots counted on 6 sections from 3 mice). By contrast strong cellular staining—presumably lytic infection—was confined (78/78 cells) to CD68^+^ macrophages (Fig. 7A). Therefore incoming virions bound to type 1 AECs but infected macrophages. Similarly 94.0 ± 5.1% of inhaled replication-deficient MuHV-4 virions associated with PDP^+^ cells (ORF50^-^, Fig. 7B, >300 dots on 6 sections from 6 mice).

In mice given WT eGFP^+^ MuHV-4, 109/113 lytically infected cells (96.5%) were eGFP^+^ and 109/140 eGFP^+^ cells (77.9%) were strongly lytic antigen^+^ (5 sections from 3 mice) (Fig. 7C). Therefore eGFP expression identified most lytically infected cells, plus eGFP^+^lytic antigen^-^ cells that were presumably latently infected. Only 8.7 ± 5.9% of antigen^+^ dots (virions) were associated with eGFP^+^ cells. Therefore most of the cells binding input virions remained uninfected.

In mice given eGFP^+^ORF50^-^ MuHV-4, the lack of new lytic infection allowed us to identify diffusely antigen^+^ cells as those accumulating input virions (Fig. 7D). 32/35 antigen^+^ cells (91.4%) were eGFP^+^, and 32/46 eGFP^+^ cells (69.6%) were antigen^+^ (8 sections from 4 mice). The greater number of eGFP^+^ cells reflected presumably that detectable eGFP expression could come from a single infecting virion, but only cells accumulating multiple virions would be detectably antigen^+^. Nonetheless antigen accumulation and eGFP expression showed a significant correlation (p<0.0001 by Fisher's exact test). By contrast only 7.1 ± 3.8% of extracellular virions (isolated antigen^+^ dots) were associated with eGFP^+^ cells. Therefore type 1 AECs bound most input virions, but alveolar macrophages then accumulated the virions and became infected, consistent with their function of collecting particulate alveolar antigens [52].

MuHV-4 infection is endocytic [53] and virion antigens did not accumulate inside PDP^+^ cells. Therefore poor virion endocytosis seemed a likely explanation for type 1 AEC non-infection. Consistent with this idea, i.n. Herpes simplex virus type 1, which binds to heparan but penetrates most cells at the plasma membrane [54], showed abundant early type 1 AEC infection without marked macrophage involvement (S5 Fig).

### Neutralizing virions for host entry

**Fig 8:**
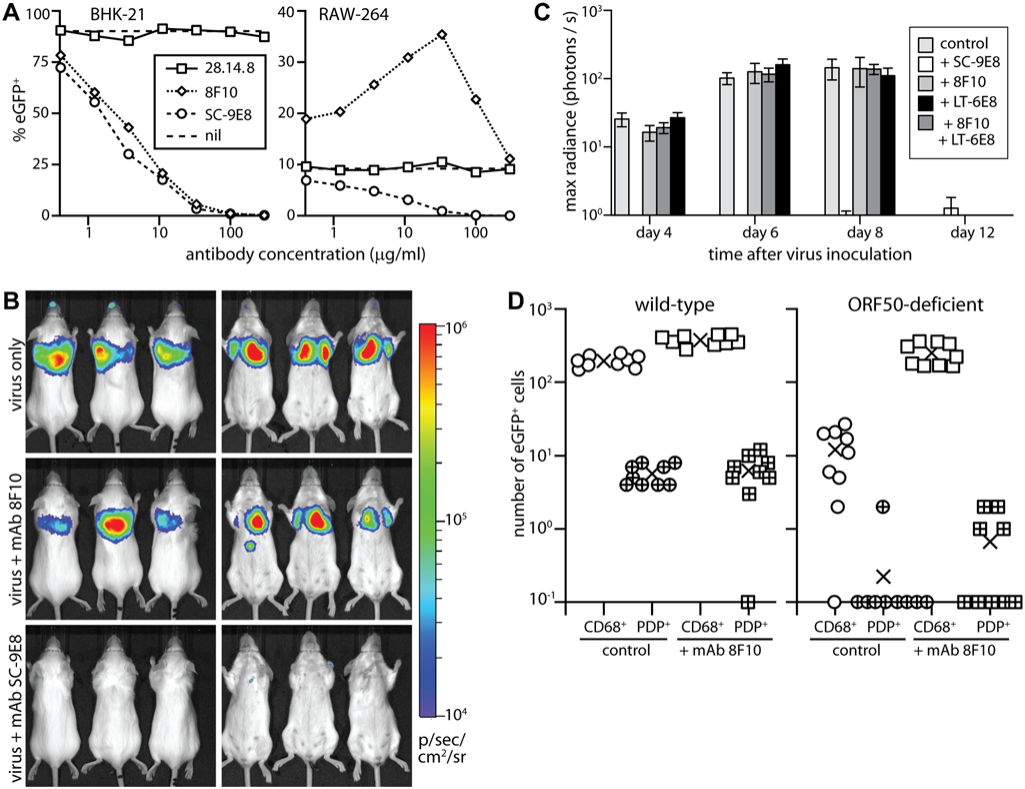
Neutralization of lung infection. **A.** EGFP^+^ MuHV-4 (EF1α-eGFP) was incubated with mAb SC-9E8 (IgG_2a_, anti-gB), 8F10 (IgG_2a_, anti-gH/gL) or 28.14.8 (IgG_2a_, anti-mouse H2D^b^, negative control). nil = no antibody. After 2h at 37°C, each virus preparation was split between BHK-21 fibroblasts (1 p.f.u./cell) and RAW-264 monocytes (5 p.f.u./cell). The cells were cultured overnight with phosphonoacetic acid (100μg/ml) to prevent lytic spread, then analyzed for eGFP expression by flow cytometry. Each point shows %eGFP^+^ of 10,000 live cells. 8F10 significantly increased RAW-264 infection at each dilution and SC-9E8 significantly reduced it (p<10^-6^ by Fisher's exact test, counting eGFP^+^ and eGFP^-^ cells for 8F10 and the negative control). **B.** Luciferase^+^ MuHV-4 was incubated or not (virus only) with mAb SC-9E8 or 8F10 (100μg/10^5^ p.f.u.), then given i.n. (10^3^ p.f.u.) to BALB/c mice. Infection was tracked by live imaging. The images show example mice at day 4, with luciferase signals in color. **C.** Luciferase^+^ MuHV-4 was incubated as in b with SC-9E8, 8F10, and no antibody (control) or with mAb LT-6E8 (IgG_2b_, anti-gp70) or LT-6E8 + 8F10. Bars show mean ± SEM of 6 mice per group tracked serially by live imaging. At days 4, 6 and 8, SC-9E8-coated virus gave significantly lower signals than the controls (p<10^-6^ by Student’s 2-tailed unpaired t test). The signals with 8F10, LT-6E8, and 8F10 + LT-6E8 were no different to the control (p>0.3). **D.** ORF50^+^ or ORF50^-^ eGFP^+^ MuHV-4 was coated or not (control) with mAb 8F10 (100μg/10^5^ p.f.u.) then given i.n. (10^4^ p.f.u.) to C57BL/6 mice. 1 day later lung sections were stained for eGFP, PDP and CD68. EGFP^+^ cells co-localizing with either cell marker were counted on 9 sections from 3 mice. Crosses show means, other symbols show individual section counts. Incubation with mAb 8F10 increased significantly the number of eGFP^+^CD68^+^ cells for both WT and ORF50^-^ viruses (p<10^-5^) but not the number of eGFP^+^PDP^+^ cells (p>0.3).

The γ-herpesviruses transmit from immune carriers and elicit salivary antibody responses [55]. Therefore shed virions may carry glycoprotein-specific antibodies. Also virions entering immune hosts or vaccinees could acquire antibodies before reaching mucosal surfaces. Therefore how bound antibodies affect host entry is important to understand. *In vitro*, immune sera inhibit MuHV-4 epithelial and fibroblast infections, but fail to inhibit and even enhance myeloid infection due to productive IgG Fc receptor (FcR)-mediated uptake [56]. This reflects that sera neutralize *in vitro* mainly by blocking cell binding, for which FcR binding can substitute. We tested 2 MuHV-4 glycoprotein-specific mAbs for their effect on lung infection: 8F10, which binds to gH/gL and blocks its interaction with heparan [57], and SC-9E8, which binds near the gB fusion loops and blocks membrane fusion [58] (Fig. 8).

*In vitro* both mAbs inhibited fibroblast infection, but while SC-9E8 inhibited RAW-264 monocyte infection 8F10 enhanced it (Fig. 8A). Enhancement reflects that FcR binding improves significantly cell-free MuHV-4 attachment to RAW-264 cells [56]. *In vivo* virion capture via type 1 AECs was efficient already, so here mAb 8F10 simply failed to neutralize (Fig. 8B, 8C). By contrast mAb SC-9E8, which neutralizes independent of cell binding [58], was effective at blocking host entry. Most transformed epithelial cell lines display both sulfated and unsulfated heparan and are bound by both gp70 and gH/gL [33]. However only gH/gL binds well to unsulfated heparan (10E4^-^NAH46^+^) [17], as displayed by the apical alveolar epithelium (Fig. 6). Accordingly, an additional block of gp70 heparan binding by mAb LT-6E8 [33] did not affect host entry (Fig. 8C).

**Fig 9:**
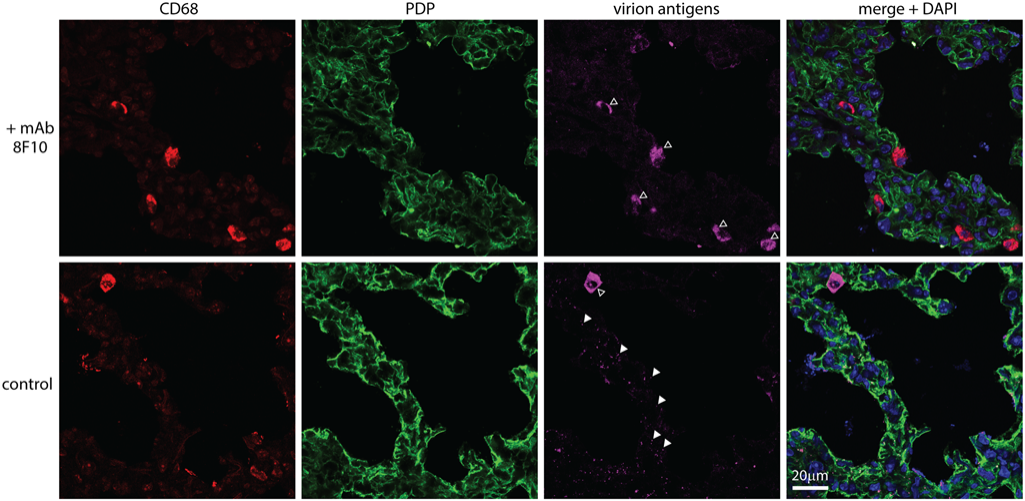
An antibody-mediated block to heparan binding inhibits MuHV-4 interaction with alveolar epithelial cells but not macrophages. MuHV-4 virions were incubated with mAb 8F10 (100μg / 10^5^ p.f.u.) or not (control) (2h, 26°C), then given i.n. to C57BL/6 mice (10^4^ p.f.u.). 5h later lung sections were stained for CD68 (red, alveolar macrophages), PDP (green, type 1 AECs) and virion antigens (cyan). Open arrows show cells accumulating virion antigens. All these were CD68^+^, regardless of antibody treatment (>50 cells counted from at least 3 sections of 2 mice). Antigen accumulation was highly variable between sections, so while higher with 8F10 the difference was not statistically significant. Filled arrows show isolated virions. Without antibody there were 70.0 ± 4.7 isolated virions per section (mean ± SEM, 6 sections), of which 96% were associated with PDP^+^ cells. With antibody there were 6.7 ± 1.4 per section, of which 67.5% were associated with PDP^+^ cells. Therefore antibody significantly reduced virion binding to epithelial cells (p<10^-6^ by Student's 2-tailed, unpaired t test) without affecting their accumulation by macrophages.

To understand better the *in vivo* fate of 8F10-opsonized virions, we exposed WT and ORF50^-^ eGFP^+^ MuHV-4 to mAb 8F10 or not, gave them i.n., and 1 day later stained lung sections for eGFP expression (Fig. 8D). Pre-incubation with 8F10 increased significantly the number of eGFP^+^ lung macrophages. Epithelial infection was unaffected, consistent with this occurring via macrophages rather than directly. Pre-incubating WT MuHV-4 with mAb 8F10 or not and staining lungs for virion antigens 5h after i.n. inoculation (Fig. 9) showed that 8F10 reduced significantly the virion binding to PDP^+^ cells without compromising their accumulation in CD68^+^ cells. Therefore blocking cell binding with antibody was insufficient to block host entry, because it merely diverted virions to where they were already going: macrophages.

## Discussion

How rhadinoviruses first infect new hosts has been little explored. Even for lymphocryptoviruses there is considerable uncertainty. *In vitro* analyses have focussed on epithelial cell and lymphocyte infections. However MuHV-4, which enters the upper respiratory tract via olfactory neurons, entered the lungs via alveolar macrophages. Most entry models envisage a target cell with a specific binding receptor that is infected readily *ex vivo*. Alveolar macrophages lacked the key initiator of MuHV-4 binding—heparan—and were infected poorly *ex vivo*. These data suggest a new paradigm, in which virions with a sophisticated fusion machinery can exploit normal host pathways for entry. One such pathway appeared to be particle scavenging by alveolar macrophages; another was opsonization-dependent endocytosis. Thus, *in vivo* neutralization had to act post-binding. As immune sera neutralize MuHV-4 mainly by blocking cell binding [56], this explains their limited capacity to block host entry [59].

*In vivo* cell diversity and interaction provide a dimension lacking from most cell lines. Co-operative infections are one reflection of this and are a recurring theme in γ-herpesvirus host colonization: EBV can infect epithelial cells by transfer from B cells [60], and MuHV-4 infects olfactory sustentacular cells via binding to adjacent neurons [17]. The homeostatic interactions of macrophage with other cell types [61] make them prime candidates to participate in co-operative infections. Here, epithelial heparan binding licensed virions for macrophage infection, and interaction with macrophages in turn licensed epithelial infection.

How might epithelial cell binding license macrophage infection? The MuHV-4 gp150 promotes virion release from heparan-deficient surfaces by inhibiting heparan-independent binding [24]. (Inhibition of EBV epithelial cell infection by gp350 [60] may analogously promote virion release.) Thus, initiating MuHV-4 infection requires heparan for binding and to engage gp150. A lack of heparan normally limits macrophage infection by non-opsonized, cell-free virions. If heparan is provided, or if the need for heparan is bypassed by gp150 disruption or by virion opsonization, then infection works well [26]. Therefore type 1 AECs licensed macrophage infection by their apical heparan capturing virions and engaging gp150.

How might virion interaction with myeloid cells license epithelial infection? The next step in MuHV-4 entry after binding is endocytosis. Virions lacking gL show an endocytic defect that can be reproduced by gH/gL-directed neutralization, implying that gH/gL engages a pro-endocytic ligand [57]. (This ligand is not heparan as distinct mAbs block gH/gL heparan binding and virion endocytosis.) Thus, a paucity of pro-endocytic ligands may strand heparan-engaged virions on type 1 AECs. (By contrast the olfactory epithelium shows no barrier to virion uptake after binding [17, 26].) The MuHV-4 gB and gH change conformation between endocytosis and fusion; fusion itself involves further changes [53, 62]. Myeloid-derived virions display constitutively the intermediate, post-endocytic forms of gB and gH and overcome a post-binding block to B cell infection [26]. We envisage that they overcome the post-binding block to type 1 AEC infection in the same way. The tropism-associated gB and gH conformation changes occur in early endosomes, whereas fusion occurs in late endosomes [63]. Therefore virions sorted from myeloid early endosomes to exosomes could be released in a tropism-switched form without a need for myeloid infection. This would explain how follicular dendritic cells transfer MuHV-4 from marginal zone to follicular B cells without becoming infected [27]. A similar cycling through alveolar macrophage endosomes would explain delayed type 1 AEC infection without prior macrophage infection.

Myeloid cells have roles in host colonization by several lymphotropic viruses [64, 65] including the human MuHV-4 ortholog, KSHV [66, 67]. Although MuHV-4 myeloid infection is sparse at steady state [68], inefficient *ex vivo* and not prominent in disease states, it is important for host entry, lymph node colonization [69] and systemic spread [27]. Myeloid cells engulf environmental antigens at many mucosal surfaces [70–72] and this can be extensive [73]. Therefore myeloid contributions to human γ-herpesvirus infections may be under-estimated.

The amount of antibody attached to shed virions is unknown, but salivary antibody has clear potential to affect tropism [74, 75]. Membrane fusion blocks or strong IgA responses might block host entry, because all infection requires fusion and mucosal IgA has a strong outward flux. However MuHV-4 elicits little post-binding neutralization [76] and little IgA [77]; antibody protects by effector recruitment [78] and poorly blocks host entry [59]. MuHV-4 is adapted to B cell physiology like EBV [79] but also to myeloid physiology, which encompasses the capture, transport and transfer of environmental antigens to B cells. Such adaptation is unlikely to be unique. Therefore γ-herpesvirus infection control strategies must consider not just the defensive functions of myeloid cells, but also their potential vulnerabilities.

## Materials and Methods

### Mice

C57BL/6J, BALB/c (Harlan UK or Animal Resources Centre, WA), LysM-cre [43] (Jackson Laboratories), Ai6-ZSgreen1 [44], CD11c-cre [80] and CD169-DTR mice [47] were bred in the Department of Pathology, Cambridge or the Herston Medical Research Centre, Queensland. For infection, 6–12 week old mice were anesthetized with isoflurane and virus (30μl) pipetted onto the nares, from where it was inhaled. For luciferase imaging, mice were injected i.p. with 2mg D-luciferin, anesthetised with isoflurane and monitored for light emission with a charge-coupled device camera (IVIS lumina, Caliper Life Sciences). CD169-DTR mice (CD169^+/DTR^) were depleted of CD169^+^ cells by i.p. diphtheria toxin (2μg / mouse) (Sigma-Aldrich).

### Ethics statement

Animal experiments were approved by the Cambridge University ethical review board, the UK Home Office (Project Licence 80/2538), and the University of Queensland animal ethics committee.

### Cells

BHK-21 cells (ATCC CCL-10), RAW-264 (ATCC TIB-71), 3T3–50 [15], NMuMG (ATCC CRL-1636) and 3T3-cre cells [24] were grown in Dulbecco’s Modified Eagle’s Medium with 2mM glutamine, 100IU/ml penicillin, 100μg/ml streptomycin, and 10% fetal calf serum (PAA laboratories). Macrophages were recovered from mice by post-mortem bronchial or peritoneal lavage, then adhered to tissue culture plastic (1h, 37°C) and washed in medium to remove contaminating B cells. The remaining cells were >95% CD68^+^.

### Viruses

All viruses were derived from a BAC-cloned MuHV-4 genome [81] with an HCMV IE1 promoter-driven eGFP expression cassette. In some experiments this loxP-flanked cassette was retained to identify infected cells by eGFP expression; for fluorochrome-switching [26], luciferase-expressing [15] and EF1α promoter driven eGFP viruses [82] it was removed by passage in 3T3-cre cells. ORF50^-^ MuHV-4 was grown and titered in 3T3-ORF50 cells, with ORF50 expression induced by doxycycline (1μg/ml). Herpes simplex virus type-1 (HSV-1) expressing eGFP from an HCMV IE1 promoter was grown and titered on BHK-21 cells [83]. MuHV-4 was recovered from infected cell supernatants by ultracentrifugation (13,000 x *g*, 2h). Any cell debris was removed by low speed centrifugation (500 x *g*, 5min) and filtration (0.45μm). HSV-1 was recovered from infected cells by low speed centrifugation (500 x *g*, 10min) then sonicated (3 x 1min). Cell-associated MuHV-4 was prepared as for HSV-1 by centrifugation and sonication of infected BHK-21 cells. All virus stocks were titered by plaque assay and stored at-70°C.

### Infectivity assays

Infectious virus was measured by plaque assay [24]: dilutions of virus stocks or organ homogenates were incubated with BHK-21 cells (2h, 37°C), overlaid with 0.3% carboxymethylcellulose, cultured for 2 (HSV-1) or 4 days (MuHV-4), then fixed (4% formaldehyde) and stained (0.1% toluidine blue) for plaque counting. To assay fluorochrome switching by MHV-RG, plaque assays were performed at limiting dilution in 96 well plates (12–24 wells per dilution), and plaques scored as red or green fluorescent under ultraviolet illumination after 4 days. To assay infection by eGFP expression, cells were infected with eGFP^+^ virus (2h, 37°C), cultured overnight (18h, 37°C) in the presence of phosphonoacetic acid (100μg/ml) to prevent further spread, trypsinized, washed x2 in PBS, and analysed for eGFP expression with a FACS Calibur (BD Biosciences). To assay neutralization, viruses were incubated (1h, 37°C) with dilutions of mAbs 8F10 (anti-gH/gL, IgG_2a_), SC-9E8 (anti-gB, IgG_2a_), or as a negative control 28.14.8 (anti-H2D^b^, IgG_2a_, American Type Culture Collection HB-27), then assayed for infectivity as above.

### Immunofluorescence

Organs were fixed in 1% formaldehyde / 10mM sodium periodate / 75mM L-lysine (24h, 4°C), equilibrated in 30% sucrose (18h, 4°C), then frozen in OCT. 9μm sections were air dried (1h, 23°C), blocked with 0.3% Triton X-100 / 5% normal goat serum (1h, 23°C), then incubated (18h, 4°C) with combinations of primary antibodies to eGFP (rabbit pAb, Abcam), mCherry (rabbit pAb, Badrilla), B220 (rat mAb RA3–6B2, Abcam), CD11c (hamster mAb N418, Abcam), CD169 (rat mAb 3D6.112, Abcam), CD206 (rat mAb MR5D3, Santa Cruz), unsulfated heparan (mouse mAb NAH46, Seikagaku Corporation), sulfated heparan (mouse mAb 10E4, Seikagaku Corporation), CD68 (rat mAb FA-11, Biolegend), PDP (goat pAb, R&D Systems), surfactant protein C precursor (goat pAb, Santa Cruz Biotechnology), MAC-2 (rat mAb M3/38, eBioscience), GR-1 (rat mAb RB6–8C5, R&D Systems), myeloperoxidase (goat pAb, R&D Systems), F4/80 (rat mAb CI:A3–1, Serotec), collagen IV (rabbit pAb, Abcam), HSV-1 (rabbit pAb, Abcam), and MuHV-4 (rabbit pAb). After incubation with primary antibodies (16h, 23°C), sections were washed x3 in PBS, incubated (1h, 23°C) with Alexa633-conjugated goat anti-rat IgG pAb, streptavidin-conjugated Alexa568 and Alexa488- or 568-conjugated goat anti-rabbit IgG pAb (Invitrogen), washed x3 in PBS, and mounted in Prolong Gold + DAPI. Fluorescence was visualised with a Leica TCS SP5 or Zeiss LSM 510 confocal microscope or a Nikon epifluorescence microscope, and analysed with ImageJ.

### ELISA

MuHV-4 virions were disrupted with 0.05% Triton-X100 in 50mM sodium carbonate pH = 8.5, and coated (18h, 4°C) onto Maxisorp ELISA plates (Nalge Nunc). The plates were washed x3 in PBS / 0.1% Tween-20, blocked with 2% bovine serum albumin in PBS / 0.1% Tween-20, incubated with 3-fold dilutions of serum from MuHV-4-exposed mice (1h, 23°C), washed x4 in PBS / 0.1% Tween-20, incubated (1h, 23°C) with alkaline phosphatase-conjugated goat anti-mouse IgG-Fc pAb (Sigma Chemical Co.), washed x5, and developed with nitrophenylphosphate substrate (Sigma). Absorbance was read at 405nm (Biorad), and the presence of a MuHV-4-specific response determined by comparison with sera from age-matched, uninfected controls.

## Supporting Information

S1 Fig B cells are not a primary infection target for MuHV-4 in lungs. **a.** Lung sections of mice given i.n. (10^5^ p.f.u.) MuHV-4 expressing HCMV IE1-eGFP were immunostained 1 day later for eGFP (green in merge), B220 (B cells, red in merge) and PDP (type 1 AECs, white in merge). Nuclei were stained with DAPI (blue). The upper panels show an overview. The lower panels show the boxed region at higher magnification. No eGFP^+^ cells (>100 counted) were B220^+^ or PDP^+^. **b.** Lung sections of mice given i.n. (10^5^ p.f.u.) ORF50^-^ (replication-deficient) MuHV-4 with HCMV IE1 promoter-driven (ORF50-independent) eGFP expression were analysed 1 day later as in a. The upper panels show an overview. The lower panels show the boxed region at higher magnification. EGFP showed no co-localization with B220 or PDP (>50 cells counted). **c.** Lung sections of mice given i.n. (10^5^ p.f.u.) MuHV-4 expressing eGFP from an EF1α promoter, were analysed 1 day later as in a. The upper panels show an overview. The lower panels show the boxed region at higher magnification. EGFP showed no co-localization with B220 or PDP (>100 cells counted).
(TIF)

S2 Fig Identification of cre expression in the lungs of lysM-cre mice. **a.** Naive LysM-cre x Ai6-Zsgreen mice were analysed for cre-mediated recombination by activation of Zsgreen expression. Lung sections were stained for PDP (type 1 AECs, red in merge) and CD68 (alveolar macrophages, white in merge). Zsgreen was visualized directly (green in merge). Nuclei were stained with DAPI (blue). The upper panels show an overview. The lower panels show the boxed region at higher magnification. Arrows in the merged image show CD68^+^ cells with Zsgreen in an endosomal distribution. CD68^-^ cells expressed Zsgreen in a more uniform distribution. PDP^+^ cells were Zsgreen^-^. The images are representative of sections from 3 mice. **b.** Lungs of mice as in a were stained for surfactant protein C precursor (SP-C, type 2 AECs, red in merge) and for CD68 (white). Cells with uniform Zsgreen expression were SP-C^+^. The white arrowhead in the merged image shows an example. Some CD68^+^ cells (with endosomal Zsgreen) also contained SP-C (grey arrow), but the cells with uniform Zsgreen were SP-C^+^CD68^-^. **c.** Lungs of mice as in a were stained for the macrophage marker MAC-2 (white) and for PDP (red). Only cells with endosomal Zsgreen expressed MAC-2, consistent with their CD68 expression. **d.** Lungs of mice as in a were stained for the neutrophil marker Gr-1 (Ly6C/Ly6G, white) and for PDP (red). GR-1^+^ cells were Zsgreen^-^. **e.** Lungs of mice as in a were stained for the neutrophil marker myeloperoxidase (red). Myeloperoxidase^+^ cells were Zsgreen^-^. **f.** Quantitation of the staining for which a-e show examples gives the distribution of >200 Zsgreen^+^ cells among different lung populations (mean ± SEM for 9 sections from 3 mice). 40% were SP-C^+^ (type 2 AECs). 20% were CD68^+^ and MAC-2^+^ (alveolar macrophages). None was GR-1^+^ or myeloperoxidase (MPO)^+^. Probably most remaining Zsgreen^+^ cells were CD68^-^SP-C^+^ type 2 AECs, as the main limit on identification was weak SP-C staining: only those unequivocally SP-C^+^ were counted. Cre expression in such cells was consistent with diphtheria toxin depleting them from lysM-diphtheria toxin receptor (DTR) transgenic mice [Miyake Y, Kaise H, Isono K, Koseki H, Kohno K, et al (2007) Protective role of macrophages in noninflammatory lung injury caused by selective ablation of alveolar epithelial type II Cells. J Immunol 178: 5001–5009].
(TIF)

S3 Fig MuHV-4 does not enter lungs by infecting type 2 AECs. Lung sections of C57BL/6 mice given i.n. eGFP^+^ MuHV-4 (10^5^ p.f.u.) 1 day before were immunostained for eGFP (green in merge), surfactant protein C precursor (SP-C, red in merge) and CD68 (alveolar macrophages, white in merge). Nuclei were stained with DAPI (blue in merge). The upper panels show an overview. The lower panels show the boxed regions at higher magnification. The images are representative of >100 cells analysed on 9 sections from 3 mice. All eGFP^+^ cells were CD68^+^; while some were also SP-C^+^, no eGFP^+^ cell was SP-C^+^CD68^-^.
(TIF)

S4 Fig Late infection of type 1 alveolar epithelial cells by replication-deficient MuHV-4. Lung sections of C57BL/6 mice given i.n. ORF50^-^-eGFP^+^ MuHV-4 (10^5^ p.f.u.) 5 days before were stained for PDP (red), CD68 (white) and either viral eGFP or virion antigens green). Nuclei were stained with DAPI (blue). Virion antigens accumulated only in CD68^+^ cells (punctate staining at this magnification), while eGFP was seen in both CD68^+^ and PDP^+^ cells (ramified staining). EGFP^+^ cells were 27.8 ± 3.6 and 15.3 ± 5.2 PDP^+^ (mean ± SEM, 6 sections from 3 mice). By contrast day 1 eGFP expression was confined entirely to CD68^+^ cells (see Fig. 2).
(TIF)

S5 Fig Comparison of host entry by MuHV-4 and HSV-1. Lung sections of C57BL/6 mice given i.n. HCMV IE1-eGFP^+^ MuHV-4 or HCMV IE1-eGFP^+^ HSV-1 (10^5^ p.f.u.) were stained 1 day later for CD68 (white in merge), PDP (red in merge), and either eGFP or viral antigens (green in merge). Nuclei were stained with DAPI (blue in merge). The low magnification images underestimate CD68 staining, since it is relatively weak, but make clear the different eGFP and viral antigen distributions for MuHV-4, which infected alveolar macrophages, and HSV-1, which infected type 1 AECs. The images are representative of 3 mice per group.
(TIF)
