# Supplementary figures and images for: Rhadinovirus Host Entry by Co-operative Infection
Source: PLoS Pathog. 2015 Mar 19;11(3):e1004761. doi: 10.1371/journal.ppat.1004761 (PMC4366105; doi:10.1371/journal.ppat.1004761)

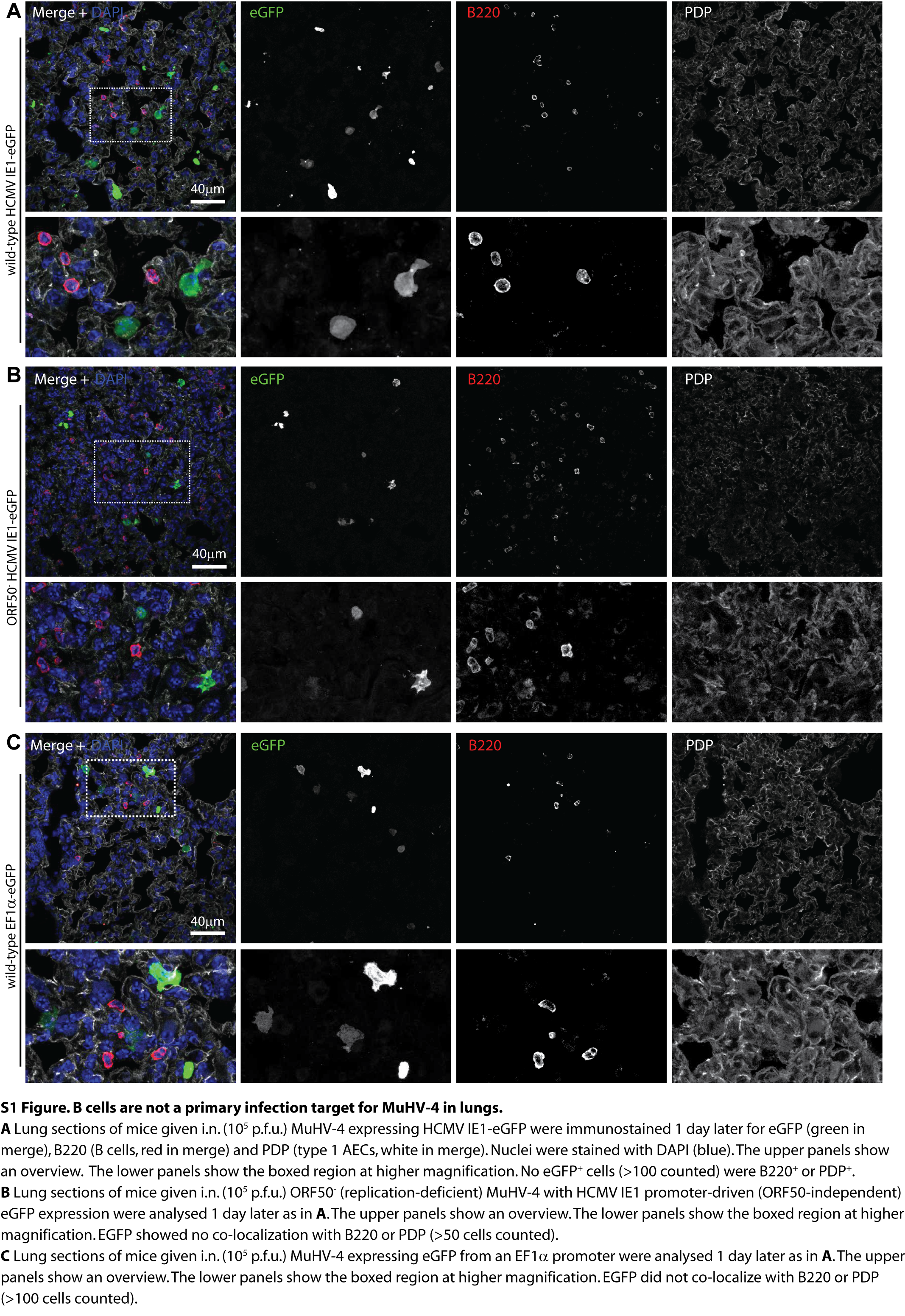

Supplement: S1 Fig — a. Lung sections of mice given i.n. (105 p.f.u.) MuHV-4 expressing HCMV IE1-eGFP were immunostained 1 day later for eGFP (green in merge), B220 (B cells, red in merge) and PDP (type 1 AECs, white in merge). Nuclei were stained with DAPI (blue). The upper panels show an overview. The lower panels show the boxed region at higher magnification. No eGFP+ cells (>100 counted) were B220+ or PDP+. b. Lung sections of mice given i.n. (105 p.f.u.) ORF50- (replication-deficient) MuHV-4 with HCMV IE1 promoter-driven (ORF50-independent) eGFP expression were analysed 1 day later as in a. The upper panels show an overview. The lower panels show the boxed region at higher magnification. EGFP showed no co-localization with B220 or PDP (>50 cells counted). c. Lung sections of mice given i.n. (105 p.f.u.) MuHV-4 expressing eGFP from an EF1α promoter, were analysed 1 day later as in a. The upper panels show an overview. The lower panels show the boxed region at higher magnification. EGFP showed no co-localization with B220 or PDP (>100 cells counted). (TIF) [file ppat.1004761.s001.tif]

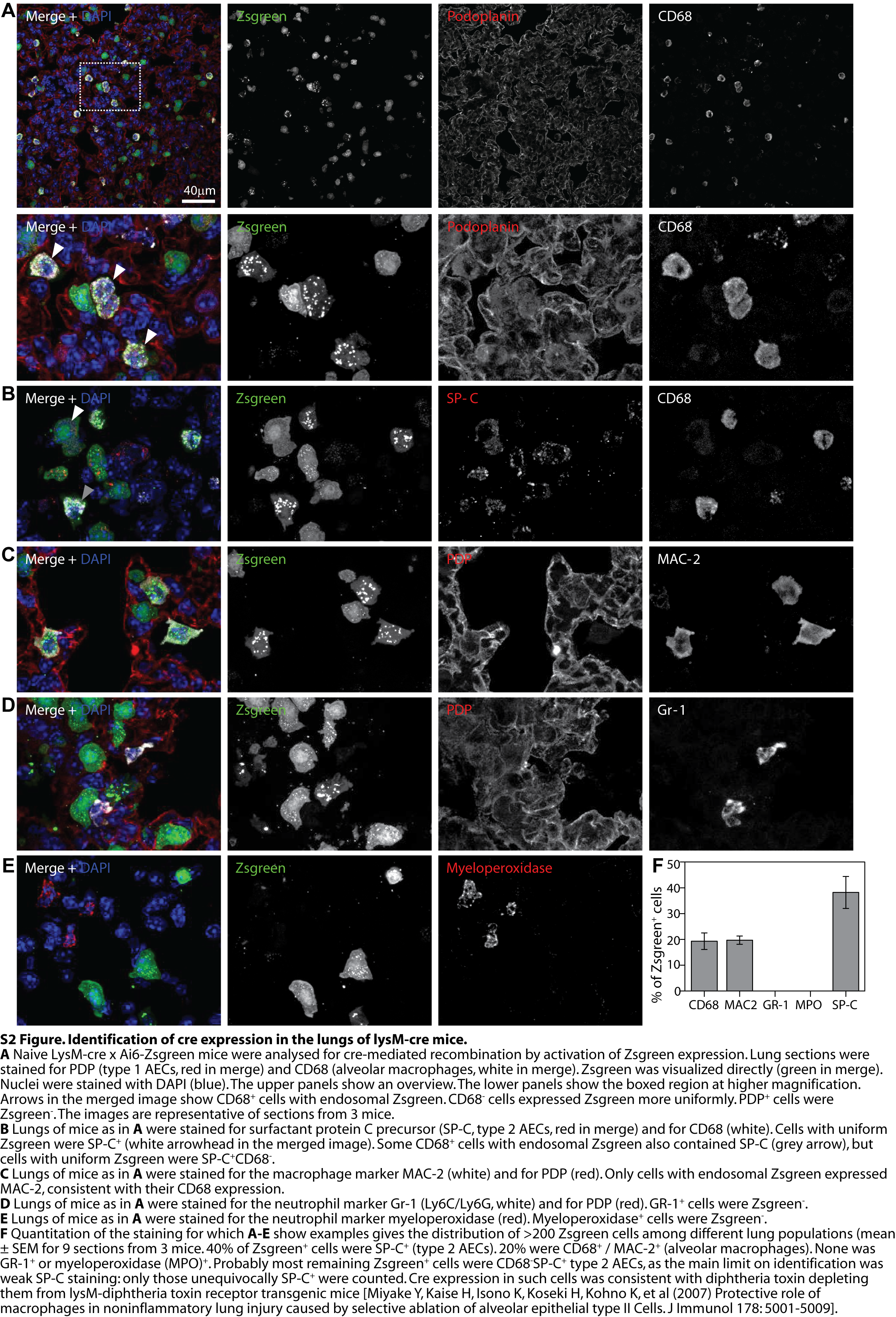

Supplement: S2 Fig — a. Naive LysM-cre x Ai6-Zsgreen mice were analysed for cre-mediated recombination by activation of Zsgreen expression. Lung sections were stained for PDP (type 1 AECs, red in merge) and CD68 (alveolar macrophages, white in merge). Zsgreen was visualized directly (green in merge). Nuclei were stained with DAPI (blue). The upper panels show an overview. The lower panels show the boxed region at higher magnification. Arrows in the merged image show CD68+ cells with Zsgreen in an endosomal distribution. CD68- cells expressed Zsgreen in a more uniform distribution. PDP+ cells were Zsgreen-. The images are representative of sections from 3 mice. b. Lungs of mice as in a were stained for surfactant protein C precursor (SP-C, type 2 AECs, red in merge) and for CD68 (white). Cells with uniform Zsgreen expression were SP-C+. The white arrowhead in the merged image shows an example. Some CD68+ cells (with endosomal Zsgreen) also contained SP-C (grey arrow), but the cells with uniform Zsgreen were SP-C+CD68-. c. Lungs of mice as in a were stained for the macrophage marker MAC-2 (white) and for PDP (red). Only cells with endosomal Zsgreen expressed MAC-2, consistent with their CD68 expression. d. Lungs of mice as in a were stained for the neutrophil marker Gr-1 (Ly6C/Ly6G, white) and for PDP (red). GR-1+ cells were Zsgreen-. e. Lungs of mice as in a were stained for the neutrophil marker myeloperoxidase (red). Myeloperoxidase+ cells were Zsgreen-. f. Quantitation of the staining for which a-e show examples gives the distribution of >200 Zsgreen+ cells among different lung populations (mean ± SEM for 9 sections from 3 mice). 40% were SP-C+ (type 2 AECs). 20% were CD68+ and MAC-2+ (alveolar macrophages). None was GR-1+ or myeloperoxidase (MPO)+. Probably most remaining Zsgreen+ cells were CD68-SP-C+ type 2 AECs, as the main limit on identification was weak SP-C staining: only those unequivocally SP-C+ were counted. Cre expression in such cells was consistent with diphtheria toxin [file ppat.1004761.s002.tif]

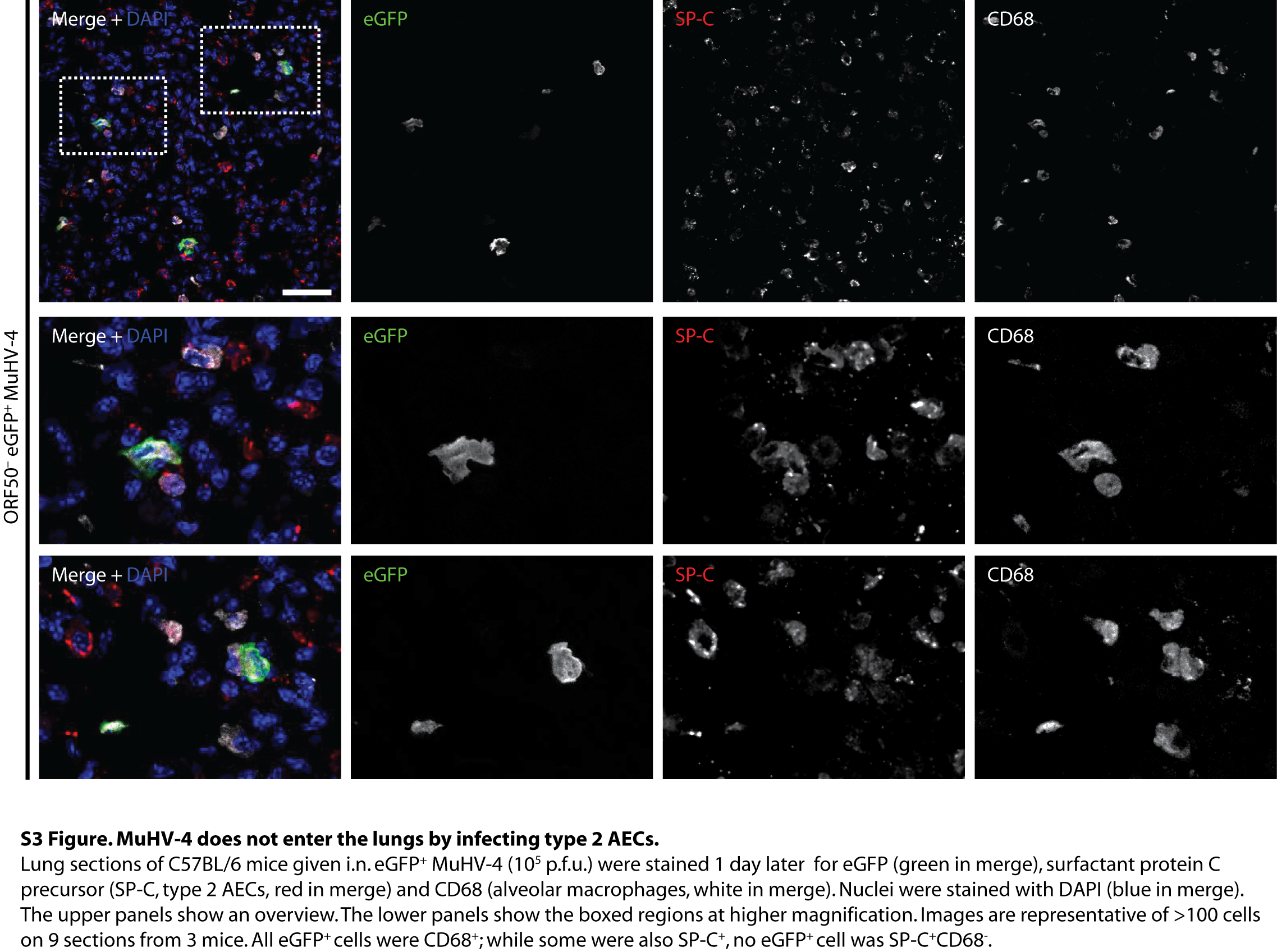

Supplement: S3 Fig — Lung sections of C57BL/6 mice given i.n. eGFP+ MuHV-4 (105 p.f.u.) 1 day before were immunostained for eGFP (green in merge), surfactant protein C precursor (SP-C, red in merge) and CD68 (alveolar macrophages, white in merge). Nuclei were stained with DAPI (blue in merge). The upper panels show an overview. The lower panels show the boxed regions at higher magnification. The images are representative of >100 cells analysed on 9 sections from 3 mice. All eGFP+ cells were CD68+; while some were also SP-C+, no eGFP+ cell was SP-C+CD68-. (TIF) [file ppat.1004761.s003.tif]

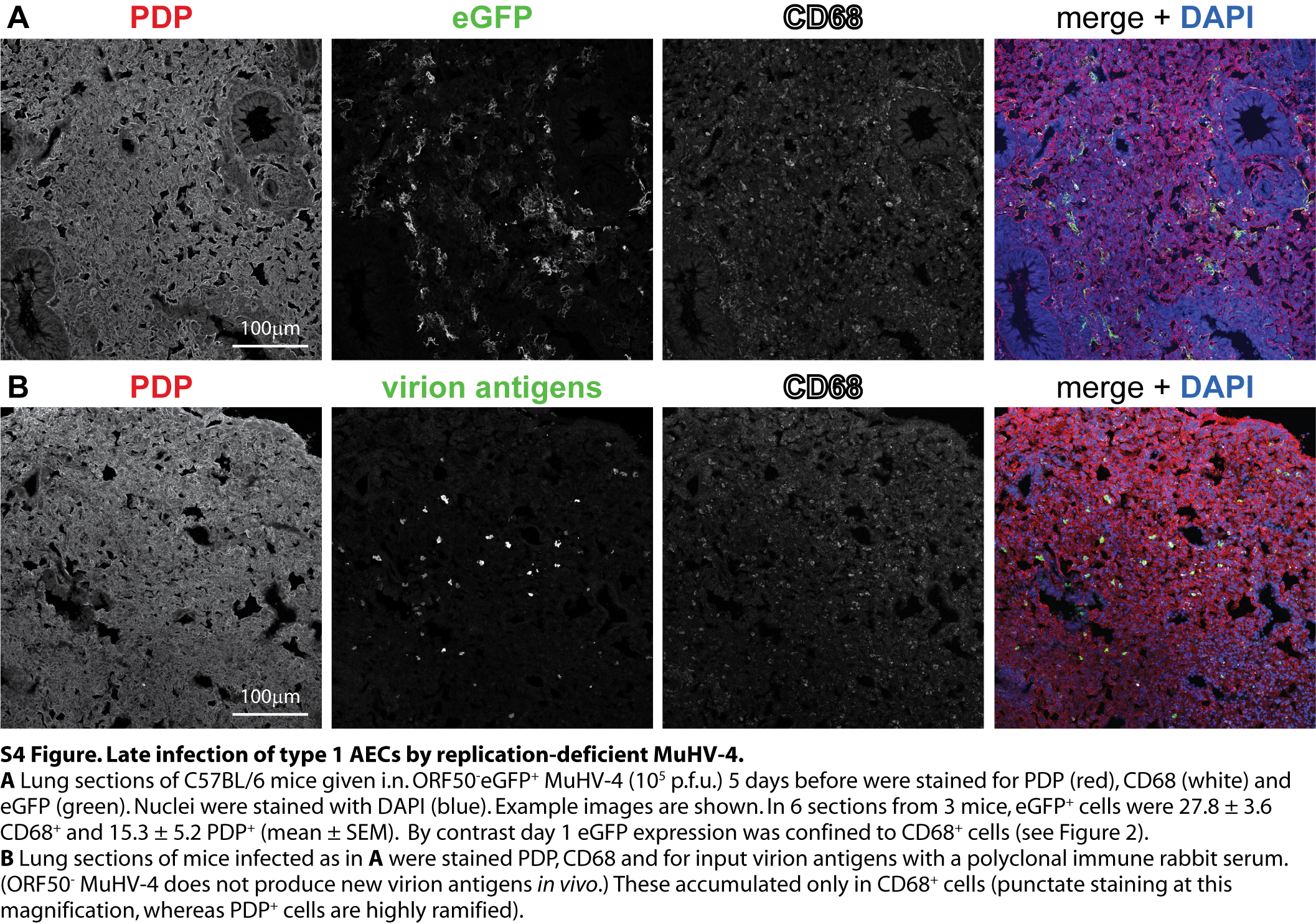

Supplement: S4 Fig — Lung sections of C57BL/6 mice given i.n. ORF50--eGFP+ MuHV-4 (105 p.f.u.) 5 days before were stained for PDP (red), CD68 (white) and either viral eGFP or virion antigens green). Nuclei were stained with DAPI (blue). Virion antigens accumulated only in CD68+ cells (punctate staining at this magnification), while eGFP was seen in both CD68+ and PDP+ cells (ramified staining). EGFP+ cells were 27.8 ± 3.6 and 15.3 ± 5.2 PDP+ (mean ± SEM, 6 sections from 3 mice). By contrast day 1 eGFP expression was confined entirely to CD68+ cells (see Fig. 2). (TIF) [file ppat.1004761.s004.tif]

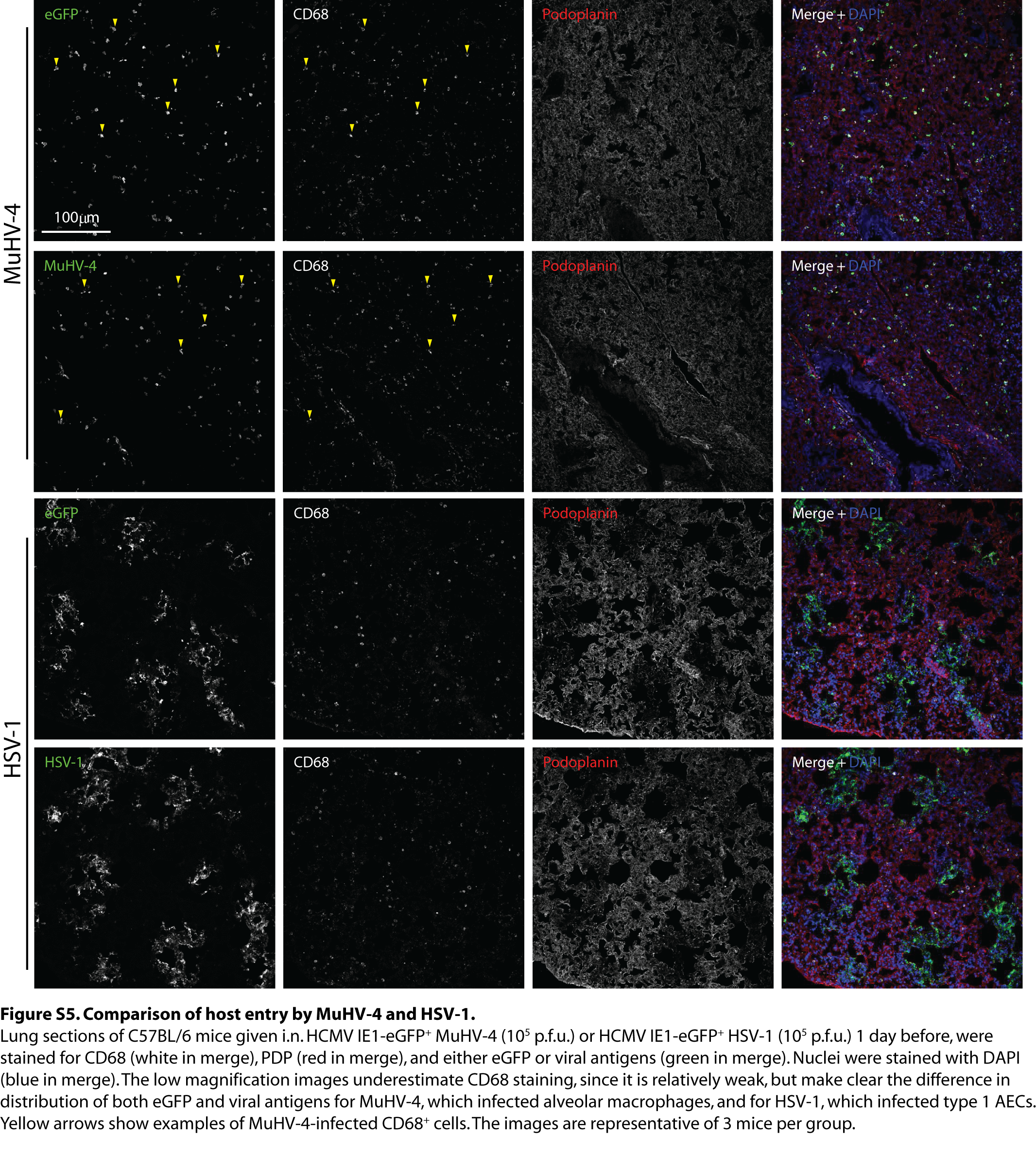

Supplement: S5 Fig — Lung sections of C57BL/6 mice given i.n. HCMV IE1-eGFP+ MuHV-4 or HCMV IE1-eGFP+ HSV-1 (105 p.f.u.) were stained 1 day later for CD68 (white in merge), PDP (red in merge), and either eGFP or viral antigens (green in merge). Nuclei were stained with DAPI (blue in merge). The low magnification images underestimate CD68 staining, since it is relatively weak, but make clear the different eGFP and viral antigen distributions for MuHV-4, which infected alveolar macrophages, and HSV-1, which infected type 1 AECs. The images are representative of 3 mice per group. (TIF) [file ppat.1004761.s005.tif]
